# Nonlinear mixed-modelling discriminates the effect of chemicals and their mixtures on zebrafish behavior

**DOI:** 10.1038/s41598-018-20112-x

**Published:** 2018-01-31

**Authors:** Patrick T. Gauthier, Mathilakath M. Vijayan

**Affiliations:** Department of Biological Sciences, University of Calgary, 2500 University Drive N.W., Calgary, T2N 1N4 Alberta Canada

## Abstract

Zebrafish (*Danio rerio*) early-life stage behavior has the potential for high-throughput screening of neurotoxic environmental contaminants. However, zebrafish embryo and larval behavioral assessments typically utilize linear analyses of mean activity that may not capture the complexity of the behavioral response. Here we tested the hypothesis that nonlinear mixed-modelling of zebrafish embryo and larval behavior provides a better assessment of the impact of chemicals and their mixtures. We demonstrate that zebrafish embryo photomotor responses (PMRs) and larval light/dark locomotor activities can be fit by asymmetric Lorentzian and Ricker-beta functions, respectively, which estimate the magnitude of activity (e.g., maximum and total activities) and temporal aspects (e.g., duration of the responses and its excitatory periods) characterizing early life-stage zebrafish behavior. We exposed zebrafish embryos and larvae to neuroactive chemicals, including isoproterenol, serotonin, and ethanol, as well as their mixtures, to assess the feasibility of using the nonlinear mixed-modelling to assess behavioral modulation. Exposure to chemicals led to distinct effects on specific behavioral characteristics, and interactive effects on temporal characteristics of the behavioral response that were overlooked by the linear analyses of mean activity. Overall, nonlinear mixed-modelling is a more comprehensive approach for screening the impact of chemicals and chemical mixtures on zebrafish behavior.

## Introduction

Zebrafish (*Danio rerio*) have proven to be an excellent model for ecotoxicological applications^1^, in part due to their tractability in laboratory settings, high fecundity, and well-understood and precisely-timed ontogeny^2^. The zebrafish is also an excellent model to study chemical effects on developmental programming, as well as early life-stage behavior^3–5^. For instance, as early as 30 hours post fertilization (hpf), zebrafish embryos exhibit a short photomotor response (PMR) involving a spike in movement over a 10 s period^4^. The PMR has high plasticity to drug exposure and has been used for high-throughput screening of neuroactive chemicals^6,7^. Also, after hatch (>72 hpf) the larvae exhibit distinct locomotor activity to alternating dark and light photoperiods^8^. Locomotor activity is supressed when larvae are held in an illuminated environment, while a switch to complete darkness evokes a spike in locomotor activity lasting around 15 min^5,8^. Recent studies suggest that this larval behavior may be disrupted by environmental contaminants^5,8,9^.

Although animal behavioral endpoints hold promise in ecotoxicology for risk assessments, they are not widely used by regulatory agencies. A major challenge in using animal behavior for risk assessment involves the wide-ranging responses that require careful assessment and selection of the most appropriate statistical analyses for biological/ecological relevance^10^. Animal activity is often measured by averaging the values temporally and/or spatially to make them amenable for linear analyses^11,12^. However, that greatly simplifies behavioral analyses because activity changes are seldom linear. This is exemplified by the nonlinear embryo PMR and larval locomotor activity profiles (i.e., asymmetric peak- and hump-shaped respectively^6,8^). These activity profiles reveal behavioral characteristics, including quickness, duration and maximum intensity, which are analogous to common parameters of nonlinear functions^13^.

Nonlinear mixed-modelling includes both fixed-effects (i.e., model parameters – phenotypical characteristics) and random-effects (i.e., within-subject effects), and allows for phenotype comparisons, while controlling for repeated measurements of activity during the behavioral trials^14,15^. Also, the inclusion of an interaction term in the fixed-effects component of the model allows for testing interactive effects^16^. Against this backdrop, our objective was to test the feasibility of using nonlinear mixed-modelling to assess the effects of chemicals either alone or as mixtures on zebrafish embryo and larval activity. Specifically, we tested the hypothesis that nonlinear mixed-modelling is a more sensitive representation of chemical effects and their interactions on early life-stage zebrafish behavior compared to linear modelling of mean activity. As a proof of concept, we carried out zebrafish embryo PMR and larval locomotor activity trials with fish exposed to model chemicals that either stimulate or suppress embryo and larvae activity. We also co-exposed embryos and larvae to a mixture of the stimulant and suppressor to demonstrate the capacity of nonlinear mixed-modelling in testing and describing mixture toxicity.

## Methods

### Zebrafish maintenance and embryo collection

Adult zebrafish (Tupfel long fin strain) were cultured in 10 L polypropylene tanks at 28.5 °C, pH 7.6, and 740 µS conductivity on recirculating systems (Pentair Aquatic Habitats, Apopka, Florida). The recirculating systems were housed in an animal care facility at the University of Calgary with a 14 h:10 h light:dark daily light cycle. Animals were fed with Ziegler^TM^ adult zebrafish diet (Pentair) and live *Artemia* (San Francisco Bay Brand, Inc, Newark, CA, USA) in the morning and evening respectively. Zebrafish were bred and the early life-stages maintained in E3 medium^17^ at 28.5 °C as described previously^5^. The animal maintenance and all experiments were approved by the animal care committee at the University of Calgary (AC17-0079) and were in accordance with the Canadian Council on Animal Care guidelines.

### Zebrafish PMR

We followed the protocol outlined in Kokel *et al*.^6^ with slight modifications. We 3D-printed a custom light emitting diode (LED)-array for use with a ZebraBox behavioral acquisition system (Viewpoint Life Sciences, Montreal, QC, Canada) that allowed for multiple configurations of LEDs (Super Bright LEDs, St. Louis, Missouri, USA) that could be activated with a remote switch. PMR trials were 30 s in duration and were carried out in total darkness, with the exception of two 1 s light pulses at 10 and 20 s^6^, and were recorded at 30 frames per second. Embryos were dechorionated at 24 hpf in 1 g L^−1^ Pronase (Sigma) and transferred (6 embryos per well) to the center 48 wells of a 96-well plate (Greiner, Sigma) with 225 µL of embryo medium per well. Only the center 48 wells were used to maximize magnification and resolution of video acquisition for activity measurements. Preliminary work identified that exposure to the β-adrenergic receptor agonist, isoproterenol, and ethanol stimulated and supressed the PMR, respectively. The treatments included 100 µM isoproterenol, 2% ethanol or a combination of both and the exposures began 30 min before each PMR trial. Final well volume was 300 uL following additions of chemical stocks. Treatments were randomly assigned by plate column, and each plate contained 2 columns (i.e., 16 wells) of control, isoproterenol, ethanol, and isoproterenol-ethanol treatments. A total of 360 embryos per treatment were used for the PMR experiments. The PMR trials began at 32 hpf with 20 min dark acclimation periods between plates. Embryo activity was quantified as Δ pixel intensity from each frame.

### Zebrafish larval locomotor activity

Larvae were transferred to each well of a 96-well plate at 80 hpf along with 225 µL of embryo medium and maintained overnight at 28.5 °C. Preliminary work identified that exposure to isoproterenol and serotonin suppressed and stimulated larval locomotor activity, respectively. The next day each well received either 20 µM isoproterenol, 100 µM serotonin or a combination of both and each plate contained 24 wells/larvae per treatment (a total of 3 plates). Final well volume was 300 uL following additions of chemical stocks. Immediately following the chemical exposure, plates were transferred to the Zebrabox and behavioral trials commenced as described previously^18^, following the lighting regime of Emran *et al*.^8^. The Zebrabox system includes backlighting in the visible spectrum from 0% to 100% intensity. Our lighting regime included 30 min at 0% intensity (i.e., dark adaptation), 30 min at 100% intensity, and a final 30 min at 0% intensity. Larval activity was calculated as total distance travelled every 30 s as described previously^5,18^.

### Nonlinear mixed-modelling of behavioral data

#### Embryo PMR

We chose an asymmetric Lorentzian function^19^ because the zebrafish embryo PMR resembles an asymmetric peak^6^. The asymmetric Lorentzian function is behaviorally relevant as it predicts activity (*L*_*(x)*_) as a function of time (*x*), duration of the excitatory period (*x*_*max*_), duration of the PMR (γ_0_), total activity (*A*), and asymmetry in excitatory and relax periods of the PMR (*a*; Supplementary Fig. S1), and is described in the following equation:

1 $${L}_{(x)}=[(2A)/(\pi {\gamma }_{(x)})]/(1+4{[(x-{x}_{max})/{\gamma }_{(x)}]}^{2}),$$

where; γ_(*x*)_ is the corrected asymmetric peak width given by:

2 $${\gamma }_{(x)}=2{\gamma }_{0}/[1+\exp [a(x-{x}_{max})]],$$

where; γ_0_ is the full width of the peak at half maxima.

#### Larval locomotor activity

We applied a mixture of the beta^13^ and Ricker functions because the 30 min activity profile resembled a hump-shaped curve with asymmetry in the excitatory and relax periods (see Emran *et al*.^8^), as well as concavity in the relax period. The beta function is behaviorally relevant in this scenario as it predicts activity (*β*_*(x)*_) as a function of time (*x*), and estimates the minimum activity (*y*_*min*_) at the time of the stimulus event, the maximum activity (*y*_*max*_), the duration of the excitatory period (*x*_*max*_), and the time at the maximum rate of increase in activity (*x*_*r*_; Supplementary Fig. S1), and is described in the following equation:

3 $${\beta }_{(x)}={y}_{min}+({y}_{max}-{y}_{min})[1+({x}_{max}-x)/({x}_{max}-{x}_{r})]{[x/{x}_{max}]}^{[({x}_{max})/({x}_{max}-{x}_{r})]}$$

The Ricker function is behaviorally relevant in this scenario as it predicts activity (*ρ*_*(x)*_) as a function of *x*, *y*_*max*_, and *x*_*max*_, and is described in the following equation:

4 $${\rho }_{(x)}=({y}_{max}/{x}_{max}){{\rm{e}}}^{-x/{x}_{max+1}}$$

The Ricker-beta function includes a simple exchange rate (*f*; Supplementary Fig. S1) between the Ricker and beta functions, and is described in the following equation:

5 $${y}_{(x)}=f{\beta }_{(x)}+(1-f){\rho }_{(x)}$$

#### Statistical analyses

Embryo PMR and larval activity trials were broken down into three stages based on light stimuli. Embryo trials consisted of baseline (0 to 10 s; ES1), PMR (11 to 20 s; ES2) and refractory (21 to 30 s; ES3) stages. Larval trials consisted of the dark acclimation (0 to 30 min; LS1), light (30 to 60 min; LS2), and dark (60 to 90 min; LS3) stages. Only ES2, and LS1 and LS3 stages exhibited nonlinearity in the activity profiles and, therefore, were the only stages assessed.

Embryo PMR (ES2) and larval locomotor activity (LS1 and LS3) were modelled with equations (1) and (5), respectively, with the ‘nlme’ function of the ‘nlme’ package in R^20,21^. As *y*_*min*_ is known to be 0, it was fixed to 0 for all Ricker-beta modelling. The effect of treatments on equations (1) and (5) parameter estimates were tested against control estimates by specifying exposure treatments as fixed-effects in ‘nlme’^15,20^. Individual 96-well plates that were used for behavioral trials were specified as a random-effect within ‘nlme’. Data were also assessed with a linear mixed-model of mean activities $(\bar{y})$ during ES2, LS1, and LS3 with the ‘lme’ function of the ‘nlme’ package in R. Mixture interactions were tested by including an interaction term for the fixed-effects in linear and nonlinear mixed-models. Statistical inference regarding treatment effects was drawn using t-tests with denominator degrees of freedom estimated according to Pinheiro and Bates^15^. Model fitting performance was evaluated by observing predicted versus observed values and assessing prediction intervals of fixed-effects (i.e., 2 × root mean square error). Assumptions of normality and homogeneity of variance were confirmed with quantile-quantile plots of within-group residuals, scatter plots of standardized residuals versus fitted values, and boxplots of standardized residuals for each subject (i.e., plate by treatment; Supplementary Fig. S2)^15^. The suitability of the mixed-models were assessed by comparing predictions of random and fixed components of the models independently^15^. Results are reported in-text as % control ± s.e.m.

A model simulation was carried out on the fixed-effects components of the linear and nonlinear mixed-models to determine the statistical power of detecting treatment-induced changes in model parameters. Power curves were drawn from simulations of varying sample and effect sizes for $\bar{y}$, *y*_*max*_, *A*, *x*_*max*_, and *x*_*r*_ using the ‘powerCurve’ function of the ‘simr’ package^22^.

## Results

### Effects of neuroactive compounds on zebrafish behavior

**Figure 1:**
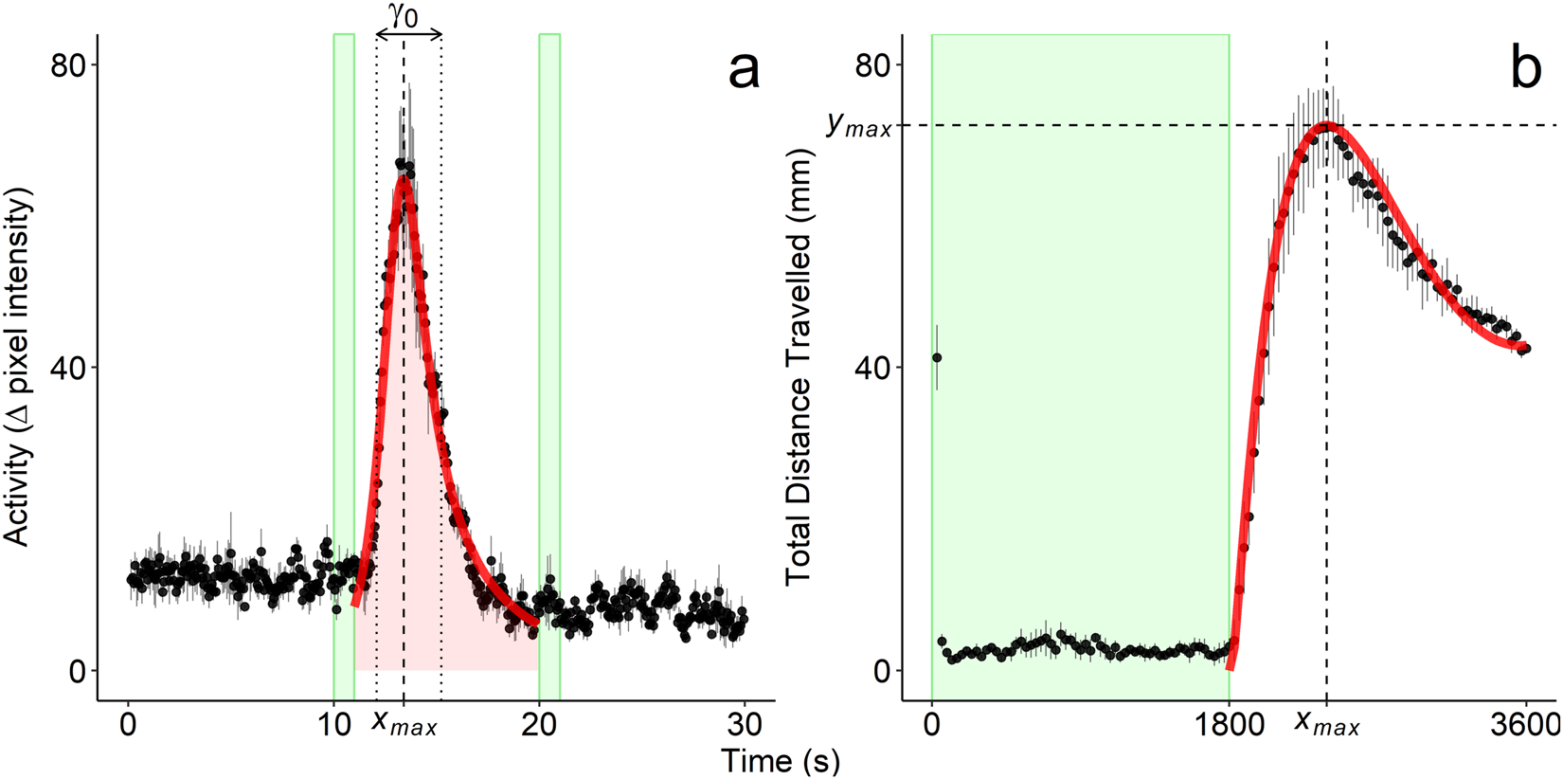
Representative activity data for the zebrafish embryo photomotor response (PMR; Panel a) and larval locomotor activity at 4 dpf (Panel b). Closed circles represent measured activity ± s.e.m. Green shading represents periods of light (Panel a and b). Red curves in Panels (a) and (b) depict predictions from asymmetric Lorentzian and Ricker-beta models, respectively. Estimated durations of the PMR (*γ*_0_) and excitatory period (*x*_*max*_) are depicted in Panel (a) by dotted and dashed lines, respectively. Red shading in Panel (a) depict estimated total embryo activity (*A*) during the PMR. In Panel (b), estimated *x*_*max*_ and maximum activity (*y*_*max*_) are depicted by vertical and horizontal dashed lines, respectively.

**Figure 2:**
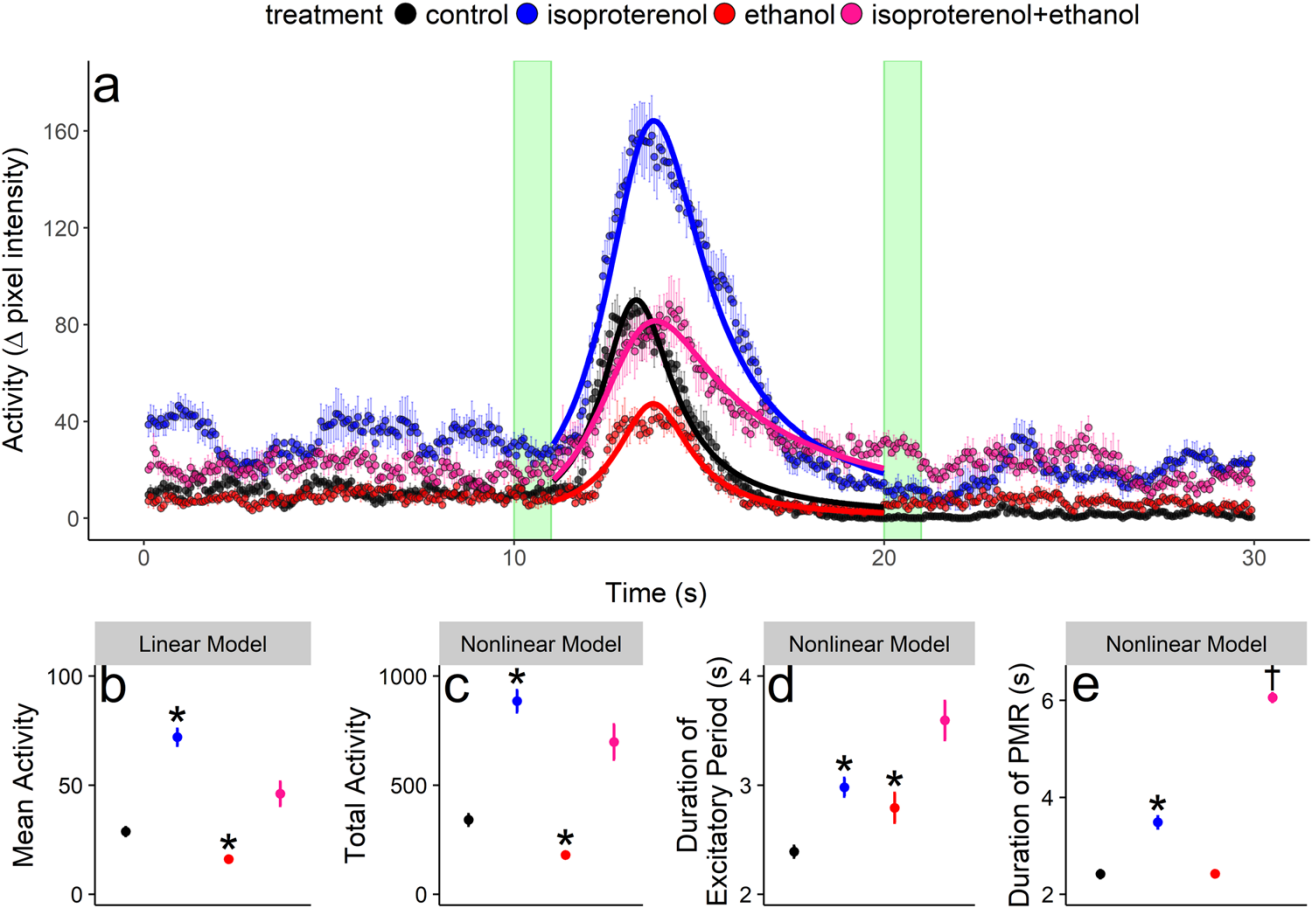
Asymmetric Lorentzian mixed-modelling results for embryo PMRs (Panel a). Colored curves in Panel (a) represent asymmetric Lorentzian model predictions for the fixed-effects of treatments. Green shaded areas represent 1 s pulses of light with an intensity of 57,295 lux. Characteristics of the behavioral phenotype, including mean $(\bar{y})$ and total (*A*) activities, and the durations of the excitatory period (*x*_*max*_) and PMR (*γ*_0_), are illustrated in Panels (b–e) (solid circles ± s.e.m.; n = 360). Asterisks indicate significant differences from control. Daggers represent significant interactive effects of the mixture.

**Figure 3:**
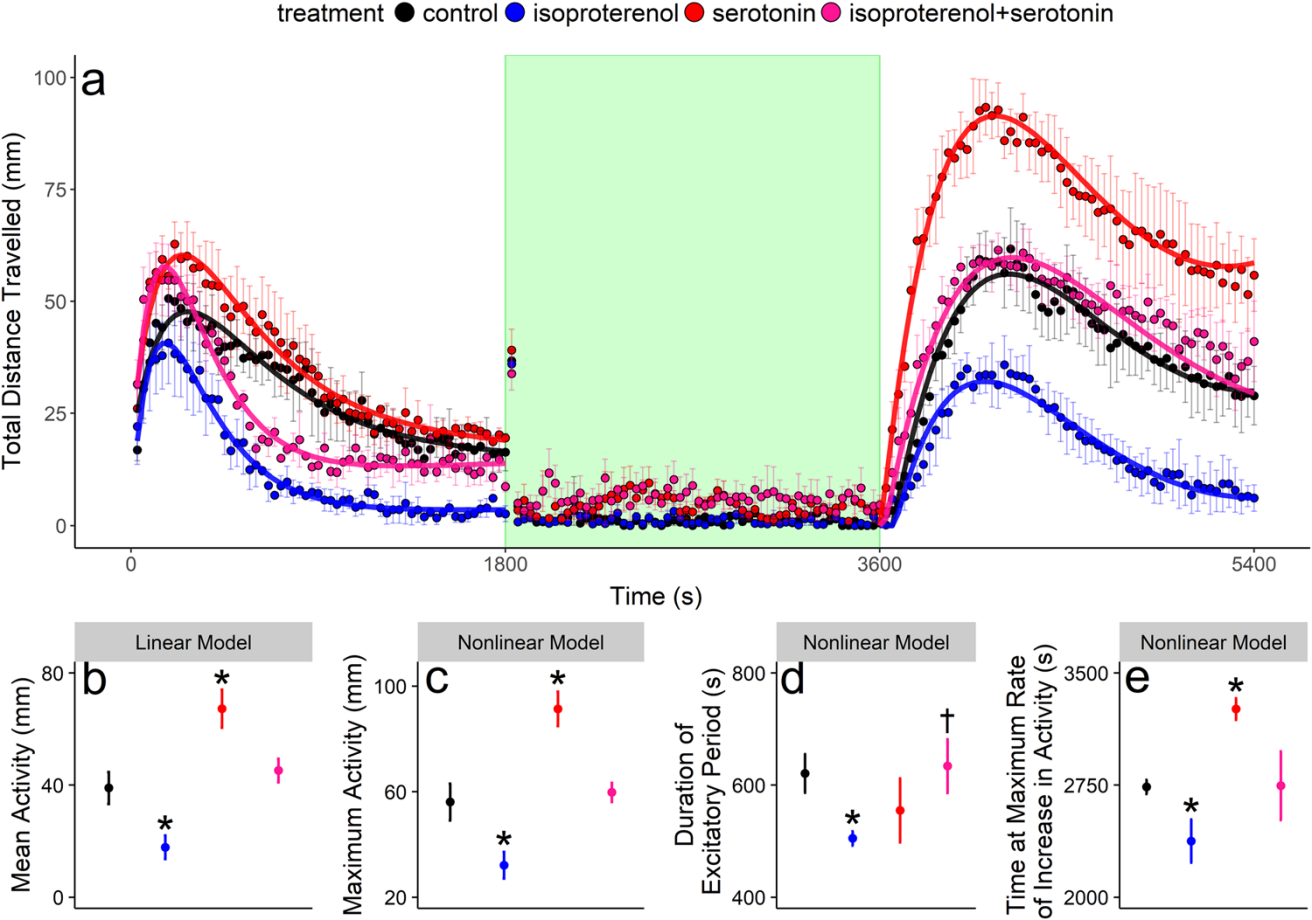
Ricker-beta mixed-modelling of larval locomotor activity (Panel a). Colored curves in Panel (a) represent the Ricker-beta model predictions for the fixed effects of treatment. The green shaded area in Panel (a) represents the light period (i.e., 1800 to 3600 s), whereas the white shaded areas represent the two dark periods (i.e., 0 to 1800 s and 3600 to 5400 s). Characteristics of the phenotype, including mean $(\bar{y})$ and maximum (*y*_*max*_) activities, duration of excitatory period (*x*_*max*_), time at maximum rate of increase in activity (*x*_*r*_) from LS3 (i.e., 3600 to 5400 s), are illustrated in Panels (b–e) (solid circles ± s.e.m.; n = 72). Asterisks indicate significant differences from control. Daggers represent significant interactive effects of the mixture.

The nonlinear mixed-models performed well in predicting the zebrafish embryo PMR and larval locomotor activity (Fig. 1), and were capable of distinguishing the chemical effects on these behavioral phenotypes (Figs 2 and 3). For both phenotypes, analysis by nonlinear mixed-modelling allowed us to unravel significant mixture interactions based on temporal characteristics of the behaviors that the linear mixed-modelling of mean activity failed to detect.

For embryo exposures, isoproterenol increased mean (150.5 ± 17.2%) and total (159.6 ± 18.7%) activities, and the duration of the PMR (44.0 ± 6.6%) and its excitatory period (24.6 ± 6.7%; Fig. 2a–e; Table 1). Exposure to ethanol decreased mean (43.9 ± 17.2%) and total (47.2 ± 18.7%) activities, while increasing the duration of the excitatory period (16.7 ± 6.7%; Fig. 2a–e; Table 1). While ethanol had no effect on the duration of the PMR, when mixed with isoproterenol, it produced a synergistic effect, increasing the duration of the PMR by 240.0 ± 32.7% (Fig. 2a,c; Table 1). For all treatments, embryos were non-responsive to the second pulse of light at 20 s (Fig. 2a).

**Table 1:** Nonlinear (nlme) and linear (lme) mixed-modelling results of zebrafish embryo photomotor responses (PMRs) following exposure to isoproterenol, ethanol, and the isoproterenol:ethanol mixture.

stage	treatment	model	parameter	estimate	se	df	t-value	p-value
ES2	control	lme	$\bar{y}$	28.75	3.51	16	8.19	<0.0001
ES2	isoproterenol	lme	$\bar{y}$	43.28	4.97	16	8.71	<0.0001
ES2	ethanol	lme	$\bar{y}$	−12.64	4.97	16	−2.55	0.022
ES2	isoproterenol:ethanol	lme	$\bar{y}$	−13.27	7.02	16	−1.89	0.077
ES2	control	nlme	*x*_*max*_	2.39	0.11	2665	21.11	<0.0001
ES2	isoproterenol	nlme	*x*_*max*_	0.59	0.16	2665	3.70	0.0002
ES2	ethanol	nlme	*x*_*max*_	0.40	0.16	2665	2.48	0.013
ES2	isoproterenol:ethanol	nlme	*x*_*max*_	0.21	0.23	2665	0.90	0.37
ES2	control	nlme	γ_0_	2.42	0.12	2665	20.57	<0.0001
ES2	isoproterenol	nlme	γ_0_	1.07	0.16	2665	6.58	<0.0001
ES2	ethanol	nlme	γ_0_	0.01	0.19	2665	0.03	0.98
ES2	isoproterenol:ethanol	nlme	γ_0_	2.57	0.35	2665	7.29	<0.0001
ES2	control	nlme	*A*	341.13	45.06	2665	7.57	<0.0001
ES2	isoproterenol	nlme	*A*	544.63	63.88	2665	8.53	<0.0001
ES2	ethanol	nlme	*A*	−161.04	63.72	2665	−2.53	0.012
ES2	isoproterenol:ethanol	nlme	*A*	−26.65	91.99	2665	−0.29	0.77
ES2	control	nlme	*a*	−0.25	0.12	2665	−2.06	0.040
ES2	isoproterenol	nlme	*a*	−0.04	0.17	2665	−0.22	0.82
ES2	ethanol	nlme	*a*	0.15	0.19	2665	0.83	0.41
ES2	isoproterenol:ethanol	nlme	*a*	−0.34	0.25	2665	−1.36	0.17

Only 11 to 20 s (ES2) of the PMR trials were assessed. Parameters represent mean activity ($\bar{y}$), duration of excitatory period (*x*_*max*_) and PMR (*γ*_0_), total activity (*A*), and peak asymmetry (*a*).

For larval behavior, exposure to isoproterenol decreased mean (54.3 ± 19.3%) and maximum (43.2 ± 12.5%) activities, the duration of excitatory period (17.9 ± 9%), and the time at maximum rate of increase in locomotor activity (52.7 ± 16%; Fig. 3a–d; Table 2). Exposure to serotonin increased mean (72.4 ± 19.3%) and maximum (62.6 ± 12.5%) activities, and the time at maximum rate of increase in activity (63.1 ± 8%; Fig. 3a,c, and e; Table 2). While exposure to serotonin had no effect on the duration of the excitatory period on its own, it ameliorated the effect of isoproterenol, decreasing its effect by 99.6 ± 44% (Fig. 3a,d; Table 2).

**Table 2:** Nonlinear (nlme) and linear (lme) mixed-modelling results of zebrafish larval locomotor activity following exposure to isoproterenol, ethanol, and the isoproterenol:ethanol mixture.

stage	treatment	model	parameter	estimate	se	df	t-value	p-value
LS1	control	lme	$\bar{y}$	29.37	3.87	8	7.58	0.0001
LS1	isoproterenol	lme	$\bar{y}$	−16.58	5.48	8	−3.03	0.016
LS1	serotonin	lme	$\bar{y}$	5.78	5.48	8	1.05	0.32
LS1	isoproterenol:serotonin	lme	$\bar{y}$	5.18	7.75	8	0.67	0.52
LS1	control	nlme	*y*_*max*_	47.68	6.40	693	7.45	<0.0001
LS1	isoproterenol	nlme	*y*_*max*_	−6.47	9.06	693	−0.71	0.48
LS1	serotonin	nlme	*y*_*max*_	13.66	9.05	693	1.51	0.13
LS1	isoproterenol:serotonin	nlme	*y*_*max*_	3.26	12.81	693	0.25	0.80
LS1	control	nlme	*x*_*max*_	271.66	21.32	693	12.74	<0.0001
LS1	isoproterenol	nlme	*x*_*max*_	−92.98	29.54	693	−3.15	0.0017
LS1	serotonin	nlme	*x*_*max*_	−24.24	29.62	693	−0.82	0.41
LS1	isoproterenol:serotonin	nlme	*x*_*max*_	11.28	41.22	693	0.27	0.78
LS1	control	nlme	*x*_*r*_	−6096.84	3545.55	693	−1.72	0.086
LS1	isoproterenol	nlme	*x*_*r*_	6512.18	3545.85	693	1.84	0.067
LS1	serotonin	nlme	*x*_*r*_	−7110.76	16814.91	693	−0.42	0.67
LS1	isoproterenol:serotonin	nlme	*x*_*r*_	11836.50	17379.06	693	0.68	0.50
LS1	control	nlme	*f*	0.39	0.06	693	6.13	<0.0001
LS1	isoproterenol	nlme	*f*	−0.32	0.08	693	−3.96	0.0001
LS1	serotonin	nlme	*f*	−0.05	0.08	693	−0.55	0.58
LS1	isoproterenol:serotonin	nlme	*f*	0.18	0.11	693	1.61	0.11
LS3	control	lme	$\bar{y}$	39.01	5.33	8	7.33	0.0001
LS3	isoproterenol	lme	$\bar{y}$	−21.19	7.53	8	−2.81	0.0080
LS3	serotonin	lme	$\bar{y}$	28.25	7.53	8	3.75	0.0056
LS3	isoproterenol:serotonin	lme	$\bar{y}$	−0.85	10.65	8	−0.08	0.94
LS3	control	nlme	*y*_*max*_	56.14	4.98	705	11.28	<0.0001
LS3	isoproterenol	nlme	*y*_*max*_	−24.24	7.04	705	−3.44	0.0006
LS3	serotonin	nlme	*y*_*max*_	35.15	7.04	705	4.99	<0.0001
LS3	isoproterenol:serotonin	nlme	*y*_*max*_	−7.02	9.96	705	−0.70	0.48
LS3	control	nlme	*x*_*max*_	623.97	39.58	705	15.77	<0.0001
LS3	isoproterenol	nlme	*x*_*max*_	−111.80	56.18	705	−1.99	0.047
LS3	serotonin	nlme	*x*_*max*_	−68.72	55.65	705	−1.23	0.22
LS3	isoproterenol:serotonin	nlme	*x*_*max*_	179.34	79.41	705	2.26	0.024
LS3	control	nlme	*x*_*r*_	−877.24	32.04	705	−27.38	<0.0001
LS3	isoproterenol	nlme	*x*_*r*_	−462.31	140.67	705	−3.29	0.0011
LS3	serotonin	nlme	*x*_*r*_	553.91	70.21	705	7.89	<0.0001
LS3	isoproterenol:serotonin	nlme	*x*_*r*_	305.12	161.97	705	1.88	0.06
LS3	control	nlme	*f*	−0.85	0.15	705	−5.65	<0.0001
LS3	isoproterenol	nlme	*f*	0.04	0.23	705	0.19	0.85
LS3	serotonin	nlme	*f*	0.62	0.21	705	2.99	0.0028
LS3	isoproterenol:serotonin	nlme	*f*	−0.22	0.31	705	−0.72	0.47

Only 0 to 1800 s (LS1) and 3600 to 5400 s (LS3) of the larval locomotor activity assays were assessed. Parameters represent mean activity ($\bar{y}$), maximum activity (*y*_*max*_), duration of excitatory period (*x*_*max*_), time at maximum rate of increase in activity (*x*_*r*_), and the proportion of the Ricker-beta function represented by Ricker and beta components (*f*).

### Suitability of nonlinear mixed-models to assess zebrafish behavior

To determine if the nonlinear models were producing accurate parameter estimates for each behavioral phenotype, we compared changes in nonlinear estimates of maximum and total activities with changes in mean activity (i.e., known values). Our rationale was that the measures of activity characteristics from the asymmetric Lorentzian, Ricker-beta, and linear models are analogous in that they all describe the general magnitude of activity of the fish during the behavioral trial. Thus, it was predicted that chemicals and their mixtures may have similar effects on these activity characteristics. Indeed this was the case, where for nonlinear and linear assessments, isoproterenol increased total and mean activity during the PMR by 159.6 ± 18.7% and 150.5 ± 17.2% respectively (Fig. 2a–c; Table 1), and decreased maximum and mean locomotor activity in larvae by 43.2 ± 12.5% and 43.9 ± 17.2% respectively (Fig. 3a–c; Table 2). The effects of ethanol and serotonin on the general magnitude of activity were also consistent between nonlinear and linear assessments, where ethanol decreased total and mean activities during the PMR by 47.2 ± 18.7% and 43.9 ± 17.2%, and serotonin increased maximum and mean locomotor activity in larvae by 62.6 ± 12.5% and 72.4 ± 19.3%, respectively. Additionally, there were no interactive effects on mean, max, or total activities associated with chemical exposures (Tables 1 and 2).

**Figure 4:**
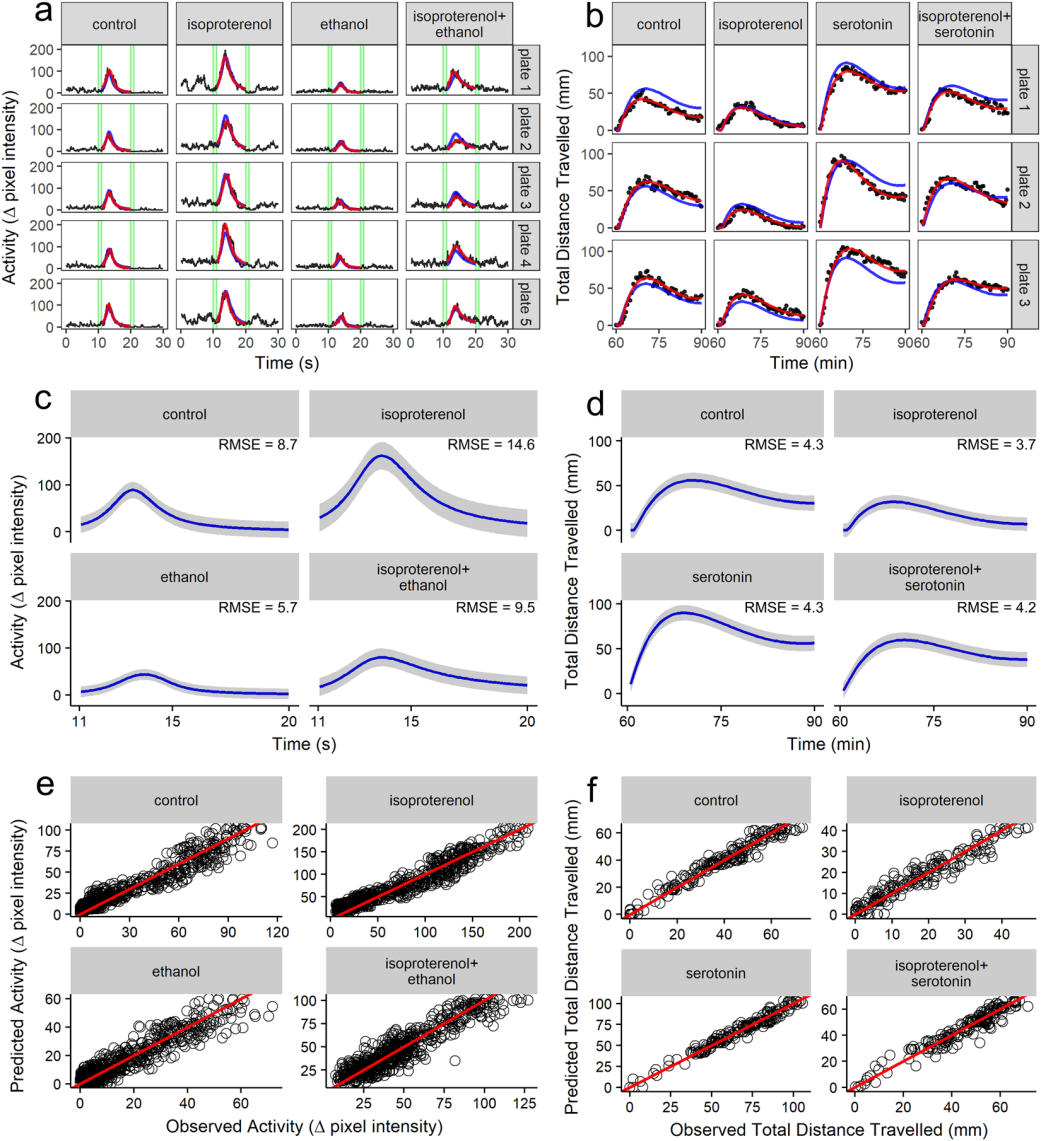
Adequacy of model fitting in terms of predicted versus observed activities and root mean square error prediction intervals. Panels (a) and (b) represent goodness of fit of the asymmetric Lorentzian and Ricker-beta mixed-models, respectively, where red curves represent within-group (i.e., random effects) predictions and blue curves represent between-group (i.e., fixed-effects) predictions. Panels (c) and (d) illustrate prediction intervals for fixed-effects (2 × root mean square errors (RMSE); grey shaded areas). Blue curves in Panels (c) and (d) are as previously described. Panels (e) and (f) illustrate the observed and predicted activities on the x- and y-axes for each treatment from the asymmetric Lorentzian (Panel c) and Ricker-beta (Panel d) models, respectively. Red lines in Panels (e) and (f) depict a 1:1 ratio of observed versus predicted values.

We then assessed the adequacy of the nonlinear mixed-models in estimating treatment effects on zebrafish behavior by comparing the treatment-level (i.e., fixed effects) and within-subject (i.e., random effects) predictions. For both phenotypes, treatment-level and within-subject effects were in close agreement (Fig. 4a,b), supporting that the nonlinear mixed-models were suitable for estimating chemical impacts on these two behavioral phenotypes. Prediction intervals for fixed-effects indicated that variability in predicted activities from both models were acceptable (Fig. 4c,d). Additionally, predicted activities closely matched observed activities from both phenotypes (Fig. 4e,f).

### Power of nonlinear mixed-models in detecting chemical impacts on behavior

**Figure 5:**
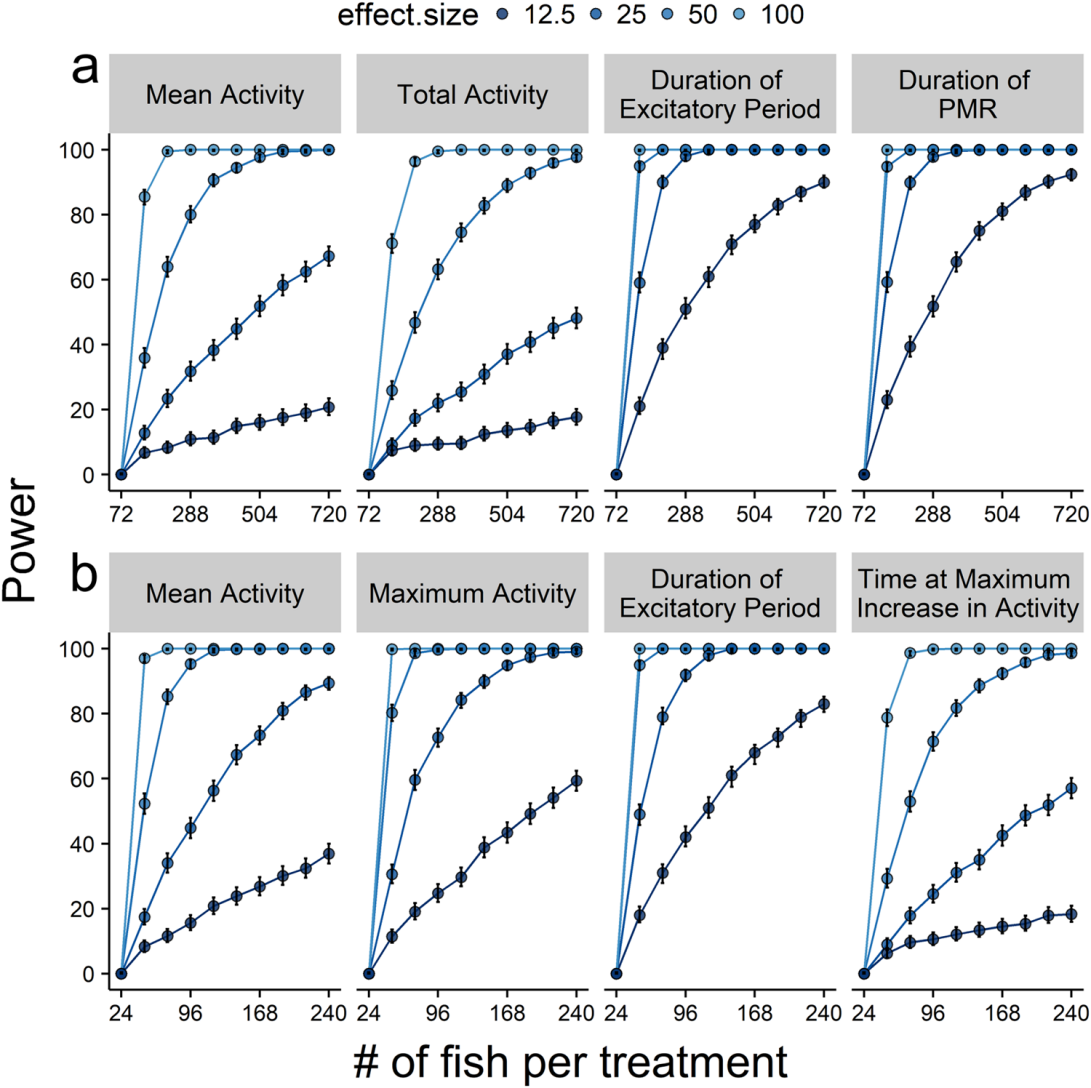
Power analyses of asymmetric Lorentzian (Panel a) and Ricker-beta (Panel b) mixed-model parameter estimates. Closed circles represent estimates of power ± 95% confidence intervals for mean activity $(\bar{y})$, total activity (*A*), maximum activity (*y*_*max*_), duration of excitatory period (*x*_*max*_) and PMR (*γ*_0_), and the time at maximum rate of increase in activity (*x*_*r*_). Effect sizes ranging between ±12.5% to 100% of control are indicated by colour shading. The y-axis represents statistical power. The x-axis represents the number of fish per treatment (i.e., samples size). A power of 80 is considered an acceptable threshold for routine detection of statistical differences.

Power analyses of the asymmetric Lorentzian mixed-model indicated the duration of the PMR and excitatory period (i.e., temporal characteristics) had the greatest power in detecting chemical-induced changes (Fig. 5a), and could routinely detect effect sizes as low at 12.5% of control with 504 to 576 embryos per treatment group (i.e., power ≥80; Fig. 5a). Comparatively, measures of the magnitude of activity (i.e., mean and total activities) had the least power in the asymmetric Lorentzian mixed-model, requiring >720 embryos per treatment to routinely detect a 25% change. For the Ricker-beta function, the duration of the excitatory period had the greatest power in detecting chemical impacts, and could routinely detect effect sizes ≥25% of control with ≥72 larvae (Fig. 5b). Mean and maximum activities performed poorer, requiring ≥192 and ≥120 larvae to routinely detect effect sizes ≥25% of control. The time at maximum rate of increase in activity performed the poorest with the Ricker-beta model (Fig. 5b).

## Discussion

We demonstrate that nonlinear mixed-modelling can be applied to estimate chemical-induced effects on zebrafish early life-stage behavior. The use of nonlinear mixed-modelling revealed chemical interactions with greater sensitivity compared to linear analyses of mean activity data. Also, the nonlinear mixed-modelling showed that chemical impact may target disparate behavioral characteristics. Our proof of concept studies underscore the utility of zebrafish embryo PMR and larval locomotor activity phenotypes for discriminating the effects of chemicals and their mixtures using the nonlinear mixed-modelling approach.

### Chemical effects on the PMR

A major difference between the nonlinear and linear approaches was that nonlinear mixed-models simultaneously assessed fundamentally disparate characteristics of behavior, including measurements of the magnitude of activity and temporal characteristics of the activity (e.g., duration of the PMR and excitatory stages), and these features were not uniformly affected by exposure to chemicals (Figs 2 and 3). The dissimilar chemical-induced effects on behavioral characteristics suggest that the changes may reflect the specific mode(s) of action of the chemicals tested. The zebrafish embryo PMR is attributed to the stimulation of opsin-based photoreceptor neurons in the hindbrain followed by a putative, yet unknown molecular signalling pathway^4^. Consequently, there are multiple potential sites of action for chemicals. The stimulation of the PMR by isoproterenol observed in our study has been well documented^6,7^, indicating an involvement of β-adrenergic signalling pathway in triggering the PMR following photoreceptor stimulation. This is in-part supported by the innervation of the zebrafish hindbrain with noradrenergic neurons^23^. Furthermore, exposure to β-adrenergic receptor antagonists eliminates the PMR^6^, and co-exposure to the β-adrenergic receptor antagonist, propranolol, completely abolishes the stimulation of the PMR by isoproterenol (Gauthier and Vijayan, Unpublished). Contrary to isoproterenol, ethanol supresses the magnitude of the PMR and this may be associated with its direct neurotoxicity to photoreceptor or motor neuron function^24–27^. Ethanol also stimulates acetylcholinesterase (AChE) activity in fish^28^, and this enzyme plays a role in terminating synaptic transmission^29^, presumably resulting in lower motor output. Thus, ethanol and isoproterenol likely affect the PMR via dissimilar modes of action and our nonlinear assessment supports this notion, whereas the linear assessment of the same dataset overlooked these dissimilarities (Fig. 2). Further work investigating the neurophysiological mechanisms that trigger and manifest the PMR (i.e., photostimulation of hindbrain photoreceptor neurons and downstream motor neuron stimulation^4^) will greatly assist in validating this concept, as well as provide further support for the use of nonlinear mixed-models in describing chemical induced effects on specific phenotypical characteristics of the PMR. This is an important consideration in terms of the advancement nonlinear mixed-modelling provides for behavioral models in the field of pharmacology and ecotoxicology, as researchers will be able to address high-throughput behavioral activity data in a more targeted and mechanistic fashion.

### Chemical effects on larval locomotor activity

The Ricker-beta model parameters describe phenotypic characteristics of locomotor activity following a change in illumination. This characterization makes the Ricker-beta model highly relevant in terms of assessing larval behavioral performance following exposure to chemicals. From an ecotoxicological perspective, locomotor activity is ecologically relevant for survival, as animals optimize their activity to minimize predation risk while maximizing resource acquisition^30^. This could explain why 4 dpf zebrafish activity is minimal when larvae are held in a bright environment they may perceive as being risky due to high visibility to a predator^31,32^, and higher when held in a dark environment they likely perceive as being comparatively safe^32–34^. In this risk model, the transition in locomotor activity between risky and safe environments can be used to assess anxiety-related disorders exacerbated or ameliorated by chemical exposure^35–37^. Less anxious fish tend to behave more boldly (i.e., having higher locomotor activity), and it has recently been shown that boldness translates to increased predation risk in the environment^38^. Thus, fish with contaminant-induced anxiolysis may be more subject to predation in the wild (i.e., more bold), and the light:dark locomotor assay can be applied as a model to assess boldness^36,37^.

As we have demonstrated, the Ricker-beta model is well-suited for analyzing data obtained from this anxiety model as it allows for the quantification of both the magnitude of activity and temporal characteristics of the behavior. Specifically, the Ricker-beta model estimates the quickness with which a fish will begin exploring a recently darkened environment through estimating the time at maximum increase in the rate of locomotor activity. The Ricker-beta model also estimates the duration of the excitatory stage during which fish increase their activity when the environment is darkened. We observed that larvae exposed to isoproterenol had lower mean and maximum activities and shorter excitatory periods following the transition from light to dark (i.e., they were behaving less boldly), and this effect was very rapid (i.e., in the order of minutes; Fig. 3; 0 to 1800 s), suggesting isoproterenol is a potent anxiogenic in 4 dpf zebrafish. This conclusion would be less convincing based solely on the linear analysis of mean activity because changes in the magnitude and temporal features of activity are not guaranteed to be uniform. Yet, while others also found that locomotor activity in larval zebrafish is negatively correlated with whole-body norepinephrine levels^39^, there has only been one case involving larval zebrafish that has directly linked decreased locomotor activity in the dark to anxiety (i.e., by measurement of thigmotaxis), but they found this using an α2-adrenoreceptor antagonist, yohimbine^40^. It appears that adrenergic receptor agonists/antagonists are important neuroactive chemicals involved in zebrafish larval anxiety.

We observed that isoproterenol and serotonin had largely opposite effects on larval locomotor activity. Serotonin modulates the locomotor activity in 4 dpf zebrafish by decreasing the duration of resting periods, resulting in a greater proportion of time being spent swimming^41^. It has also been suggested that serotonin acts to increase activity in zebrafish in response to a sudden stimulus change^42^, such as the transition from light to dark environments between LS2 and LS3. This is consistent with the finding that both maximum activity and the time at maximum rate of increase in activity increased following exposure to serotonin during LS3. Although the role of the serotonergic system in zebrafish anxiety remains unclear^42,43^, our results showing an opposite effect of serotonin compared to isoproterenol, leads us to propose an anxiolytic effect of this neuromodulator on 4 dpf zebrafish larvae. Our results suggest that nonlinear mixed-modelling can provide behaviorally meaningful results, using the light:dark zebrafish larval locomotor activity, for ecotoxicological applications.

### Mixture effects on zebrafish behavior and ecological implications

The nonlinear mixed-models were capable of detecting chemical interactions in both phenotypes, including changes in temporal characteristics of the zebrafish embryo and larval behaviors (Figs 2 and 3). Isoproterenol increased the duration of the PMR, while ethanol had no effect, and yet when combined the isoproterenol and ethanol mixture produced an increase in the duration of the PMR that could not be accounted for by their individual effects (Fig. 2). Potential explanations for this interactive effect are scarce which is exacerbated by the paucity of information regarding the underlying mechanisms that mediate the PMR. Nonetheless, acute co-exposure to ethanol stimulates the production of cyclic AMP (i.e., secondary messenger in the β-adrenergic signalling pathway) and β-endorphin release following β-adrenergic induction by isoproterenol in neurons^44^, and this may influence the potency of isoproterenol in modulating the PMR. However, there remains no explanation as to why ethanol would potentiate the effect of isoproterenol on the duration of the PMR, while having no interactive influence on other behavioral characteristics (i.e., magnitude of activity and duration of excitatory period).

For larval trials, exposure to serotonin had no effect on the duration of the excitatory period on its own, but completely abated the effect of isoproterenol (Fig. 3). Again, it is difficult to speculate as to the cause of the interaction of isoproterenol and serotonin without greater knowledge regarding the neurophysiological mechanism of larval locomotor activity. However, serotonin inhibits cyclic AMP production in neurons of the basolateral amygdala (BLA)^45,46^, a region of the brain mediating behavioral outcomes and a target for neurological disorders in mammals, including boldness and anxiety^47^. When present along with isoproterenol, serotonin and other cyclic AMP inhibitors supress post-synaptic β-adrenergic signalling in the brain^46,48,49^. Zebrafish also possess analogous structures to the BLA, and this is thought to play key roles in anxiety and boldness, among other behaviors^50^. Consequently, an inhibitory action of serotonin on isoproterenol signalling may be involved in the suppression of the anxiolytic effect of isoproterenol by serotonin in the 4 dpf zebrafish, but this remains to be elucidated.

Only through simultaneously assessing a diversity of phenotypic characteristics via nonlinear mixed-modelling were we capable of detecting interactions among the neuroactive chemicals we tested on zebrafish embryo and larval behavior. These interactive effects would have been completely overlooked if we had only assessed behavior in terms of mean activity through linear modelling. Based on these results we posit that the nonlinear approach is better suited for detecting the effects of single chemicals as well as chemical mixtures, especially given that the measures span the magnitude and temporal changes in activity. The benefits of the nonlinear versus linear approach will be subject to the chemicals that are being tested (i.e., some chemicals may only impact the magnitude of activity). However, without prior knowledge regarding the interactive and main effects of chemical mixtures, the nonlinear approach is more robust than linear analyses as it has a higher probability of detecting singular and mixture effects by simultaneously assessing a diversity of behavioral characteristics.

We present nonlinear functions to characterize two phenotypes from two different life-stages of the zebrafish. The combined capacity to assess specific characteristics of behavior, as well as agonist and antagonist interactions, leads us to propose that the asymmetric Lorentzian and Ricker-beta functions are amenable for the characterization of chemical-induced effects on early-life stage zebrafish activity. Also, there are other established nonlinear functions (see examples in Archontoulis and Miguez^13^), that may be suitable for additional activity phenotypes (e.g., freeze responses) with distinctly different lineshapes (e.g., asymptotic). Moreover, once behavioral phenotypes have been modelled, estimated parameters can be extracted for additional analyses, such as concentration-response curves to determine the effective concentrations for behavioral modulations. Acquiring effective concentrations will aid in translating chemically-induced behavioral modulation into information useful to risk assessors and regulatory agencies in environmental health applications, and pharmacologists for drug dosing applications. Combined with the clear relevance of model parameters to animal performance, we see potential for nonlinear mixed-modelling to expand the use of behavioral toxicology for risk assessment and environmental guideline development.

## Electronic supplementary material

Supplemental Information
