# Supplementary material for: Nonlinear mixed-modelling discriminates the effect of chemicals and their mixtures on zebrafish behavior
Source: Sci Rep. 2018 Jan 31;8:1999. doi: 10.1038/s41598-018-20112-x (PMC5792435; doi:10.1038/s41598-018-20112-x)
Supplement: Supplementary file 1 — Supplemental Information [file 41598_2018_20112_MOESM1_ESM.pdf]

SUPPLEMENTAL INFORMATION

Nonlinear mixed-modelling discriminates the effect of chemicals and their mixtures on zebrafish behavior

Patrick T. Gauthier[1] and Mathilakath M. Vijayan[1,*]

[1]Department of Biological Sciences, University of Calgary, Calgary, Alberta, Canada T2N1N4

[*]Corresponding author contact: matt.vijayan@ucalgary.ca

Summary of supplemental information

Supplementary Figure S1 ....S2
Supplementary Figure S2 ....S3

pp S1 – S3

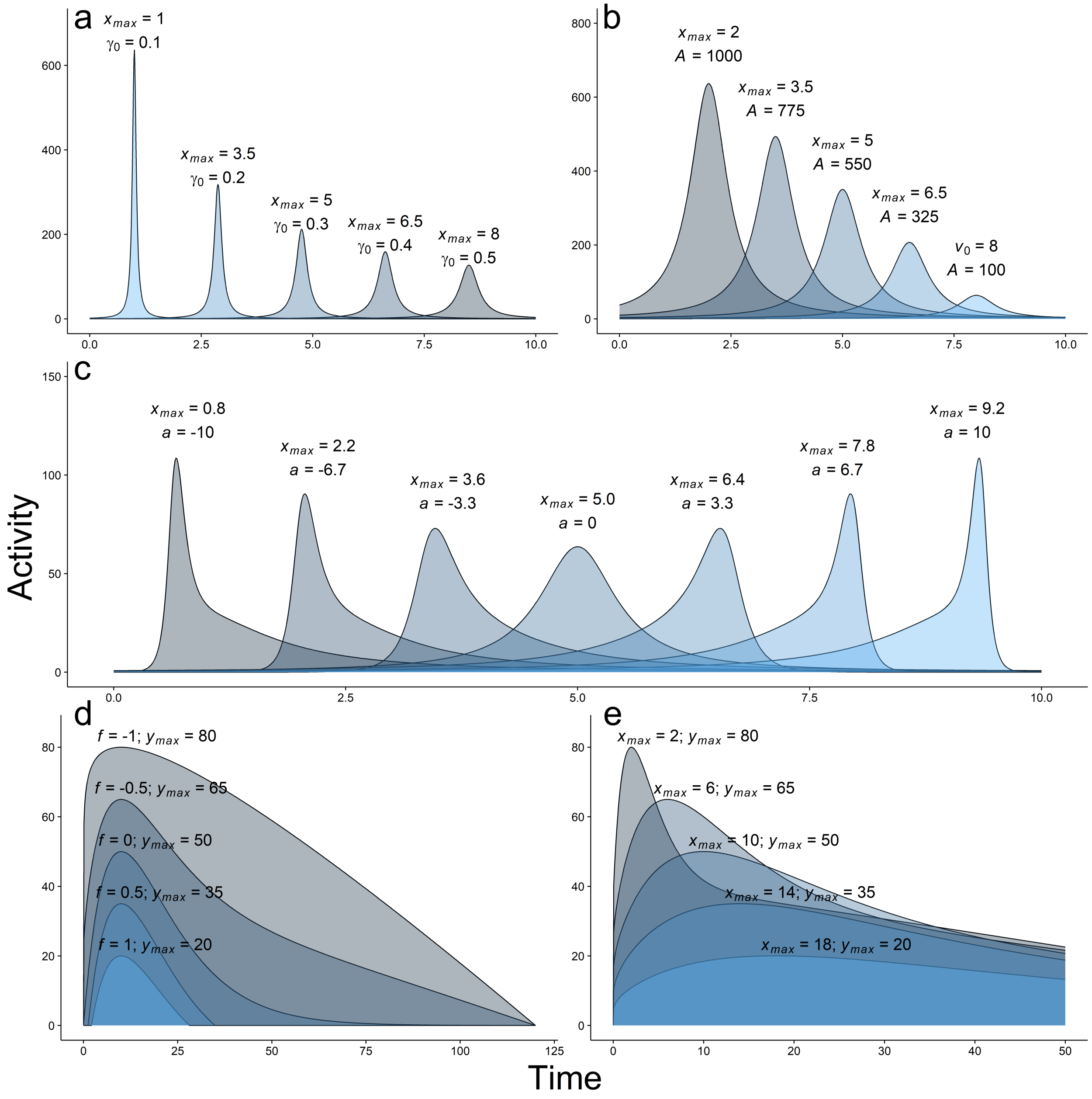

Supplementary Figure S1. Visualization of asymmetric Lorentzian (Panels a-c) and Ricker-beta (Panels d and e) functions depicting obtainable lineshapes with varying parameters. For lineshapes in Panels (a) and (b), *a* was fixed at 1. For lineshapes in Panel (c), *γ0* was fixed at 1. For all examples Ricker-beta models, y_{min} and x_r were fixed at 0 and -100 respectively (Panels d and e). For Ricker-beta lineshapes depicted in Panel (e), *f* was fixed at 0.5.

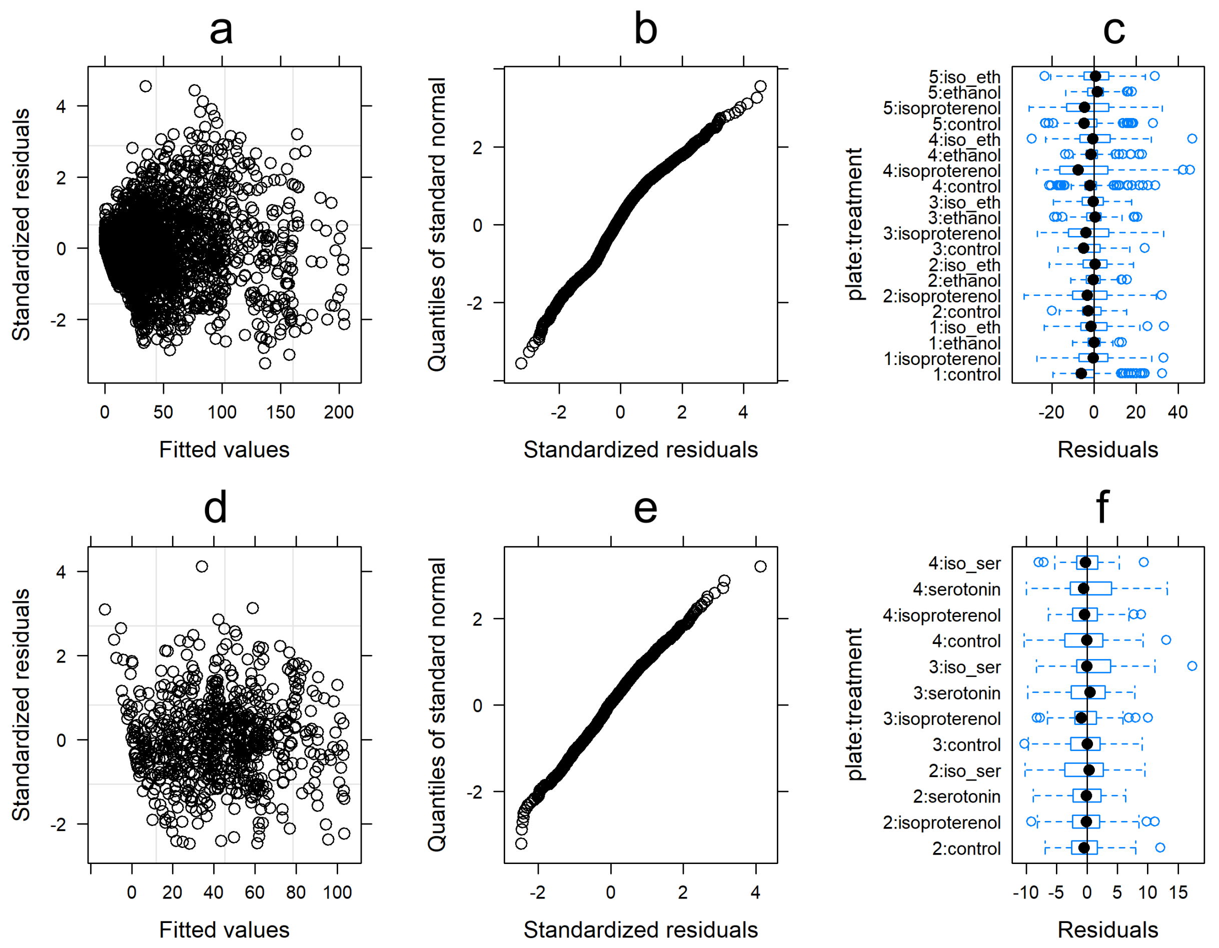

Supplementary Figure S2. Diagnostic plots for asymmetric Lorentzian (Panels a-c) and Ricker-beta (Panels d and e) mixed-models. Panels (a) and (d) illustrate homogeneity of variance of the standardized residuals versus fitted values. Panels (b) and (e) illustrate the normality of standardized residuals in comparisons to quantiles of a standard normal distribution. Panels (c) and (f) illustrate the centrality of residuals at zero and their independence from random effects (i.e., plate by treatment).
